# *In-Vitro* Detection of Small Isolated Cartilage Defects: Intravascular Ultrasound Vs. Optical Coherence Tomography

**DOI:** 10.1007/s10439-018-2073-z

**Published:** 2018-06-26

**Authors:** T. Horeman, E. C. Buiter, B. Pouran, M. Stijntjes, J. Dankelman, G. J. M. Tuijthof

**Affiliations:** 1 Department of Biomechanical Engineering, Delft University of Technology, Delft, The Netherlands; 2 Department of Orthopedic Surgery, Academic Centre for Evidence-based Sports Medicine (ACES), Academic Medical Centre, Amsterdam, The Netherlands; 3 Department of Orthopaedics, UMC Utrecht Regenerative Medicine Centre, Utrecht, The Netherlands; 4 Research Centre Smart Devices, Zuyd University of Applied Sciences, Heerlen, The Netherlands

**Keywords:** Orthopedics, OCT, IVUS, Needle intervention, Catheter imaging, (Osteo)chondral defects

## Abstract

This experimental work focused on the sensor selection for the development of a needle-like instrument to treat small isolated cartilage defects with hydrogels. The aim was to identify the most accurate and sensitive imaging method to determine the location and size of defects compared to a gold standard (µCT). Only intravascular ultrasound imaging (IVUS) vs. optical coherent tomography (OCT) were looked at, as they fulfilled the criteria for integration in the needle design. An *in-vitro* study was conducted on six human cadaveric tali that were dissected and submerged in saline. To simulate the natural appearance of cartilage defects, three types of defects were created *via* a standardised protocol: osteochondral defects (OCD), chondral defects (CD) and cartilage surface fibrillation (CSF), all sized between 0.1 and 3 mm in diameter. The detection rate by two observers for all diameters of OCD were 80, 92 and 100% with IVUS, OCT and µCT, for CD these were 60, 83 and 97%, and for CSF 0, 29 and 24%. Both IVUS and OCT can detect the presence of OCD and CD accurately if they are larger than 2 mm in diameter, and OCT can detect fibrillated cartilage defects larger than 3 mm in diameter. A significant difference between OCT–µCT and IVUS–µCT was found for the diameter error (*p* = 0.004) and insertion depth error (*p* = 0.002), indicating that OCT gives values closer to reference µCT. The OCT imaging technique is more sensitive to various types and sizes of defects and has a smaller diameter, and is therefore preferred for the intended application.

## Introduction

Ankle sprains and cartilage fractures are common. In up to 50% of all ankle sprains and fractures, isolated cartilage defects occur.^20^ These defects involve the articular cartilage and subchondral bone, which can result in deep ankle pain, stiffness of the joint and impaired movement, and if left untreated may evolve into posttraumatic osteoarthritis.^36^ Therapeutic treatments have been developed to alleviate pain, to restore functionality and to extend the time until total joint replacement.^4^,^5^,^25^ The treatments depend on the size and severity of the cartilage defect, starting with conservative treatment for International Cartilage Repair Society (ICRS) Grade I and II defects, followed by surgical treatment for ICRS Grade II–IV defects and implants for partial joint replacement.^5^,^21^,^25^,^34^ However, evidence indicates less favourable results after surgical treatment for larger and older cartilage defects with disturbed joint homeostasis^16^,^26^ and indicates some healing potential for small cartilage defects (ankle < Ø 8 mm).^9^,^12^,^15^

**Figure 1:**
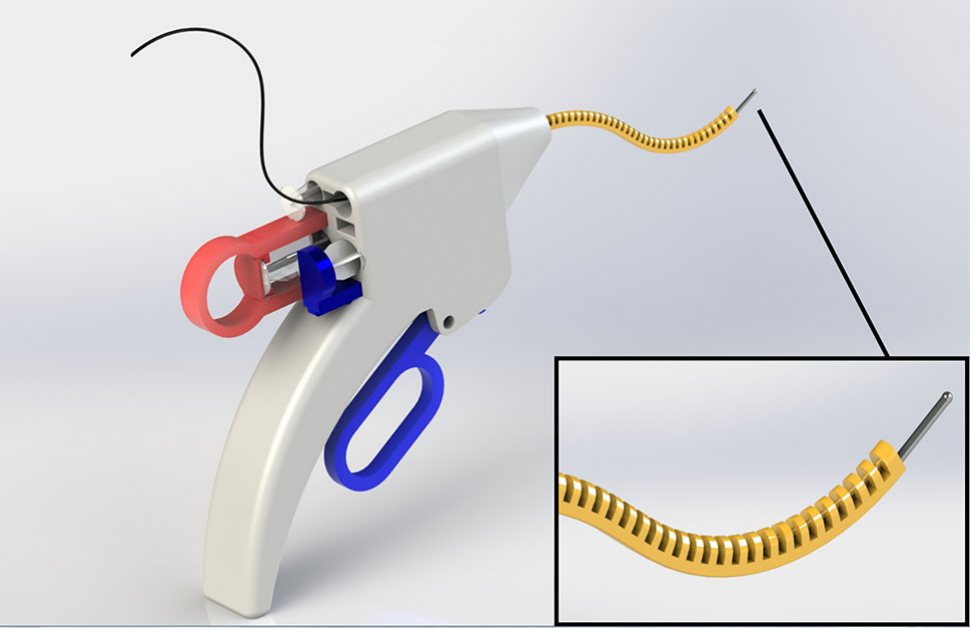
An artist’s impression of a multifunctional steerable needle device currently in development at the BioMechanical engineering department of TU Delft for the detection and treatment of articular cartilage defects in the ankle. The foreseen dimensions are similar to conventional 7- to 8-gauge needles (diameter < 5 mm).

This knowledge combined with the recent development of injectable hydrogels^2^,^13^,^17^,^29^ sets the stage for even less invasive interventions at an early stage, for example by using a needle intervention to seal small isolated cartilage defects with hydrogel. Figure 1 is an artist’s impression of a multifunctional steerable needle device currently in development for such a medical intervention. For these slender needle-like instruments, it is crucial that the needle tip finds the exact location of the isolated cartilage defect within the joint. This requires local cartilage tissue characterisation.

An overview of tissue characterisation techniques is given by Nieminen.^19^ We foresee the use of a tissue characterisation imaging system within a needle-like instrument outside the operation room in various scenarios, such as day-care clinics and, ultimately, sport fields. Therefore, the targeted imaging system should be slender, flexible, small (diameter < 2 mm) and mobile. This rules out traditional imaging techniques such as CT and MRI (not mobile^32^), as well as arthroscopes or arthroscopic characterisation techniques, such as the streaming potential method (diameters > 2 mm and rigid).^8^ Two possible methods were left that meet the design criteria: intravascular ultrasound (IVUS)^11^,^14^,^33^ and optical coherence tomography (OCT).^6^,^7^,^27^ IVUS and OCT use the reflections of sound and light, respectively, from tissue to create an image of the tissue. Both methods are clinically available. Their predominant application is in vessel imaging during coronary interventions, and in the last decade they have also been studied for application in cartilage characterisation.^11^,^14^,^33^ IVUS and OCT provided quantitative information on repaired chondral and osteochondral defects 2–8 mm in diameter in fresh bovine, equine and caprine models.^6^,^7^,^11^,^14^,^27^ In these studies, IVUS was capable of discriminating between fibrillated and intact cartilage and providing quantitative information on defect depth and surface roughness.^11^,^14^,^33^ OCT has been shown to be a reproducible technique in quantitative analysis of cartilage thickness and to a lesser extent when measuring repair tissue area and surface roughness.^1^,^6^ However, to detect and treat isolated defects at an early stage, we estimated that defect sizes as small as 1 mm in diameter and fissures 0.1 mm in width should be measured.^9^,^28^,^30^ This should be done in different human intra-articular joints, with ankle joints being challenging as the cartilage thickness is relatively small (mean of 1 mm).^1^,^28^ Furthermore, the appearance of isolated defects differs depending on whether the trauma mechanism was wear or tear, namely a complete crack with or without bone damage, bruising or fibrillation.^2^

Therefore, the aim of the present research was to assess the performance of IVUS and OCT in detecting small and different types of cartilage defects in human cadaveric tali compared to a reference micro-computed tomography (µCT), in order to select the most accurate and sensitive technique for integration in the multifunctional steerable needle device.

## Method

### Sample Preparation

**Figure 2:**
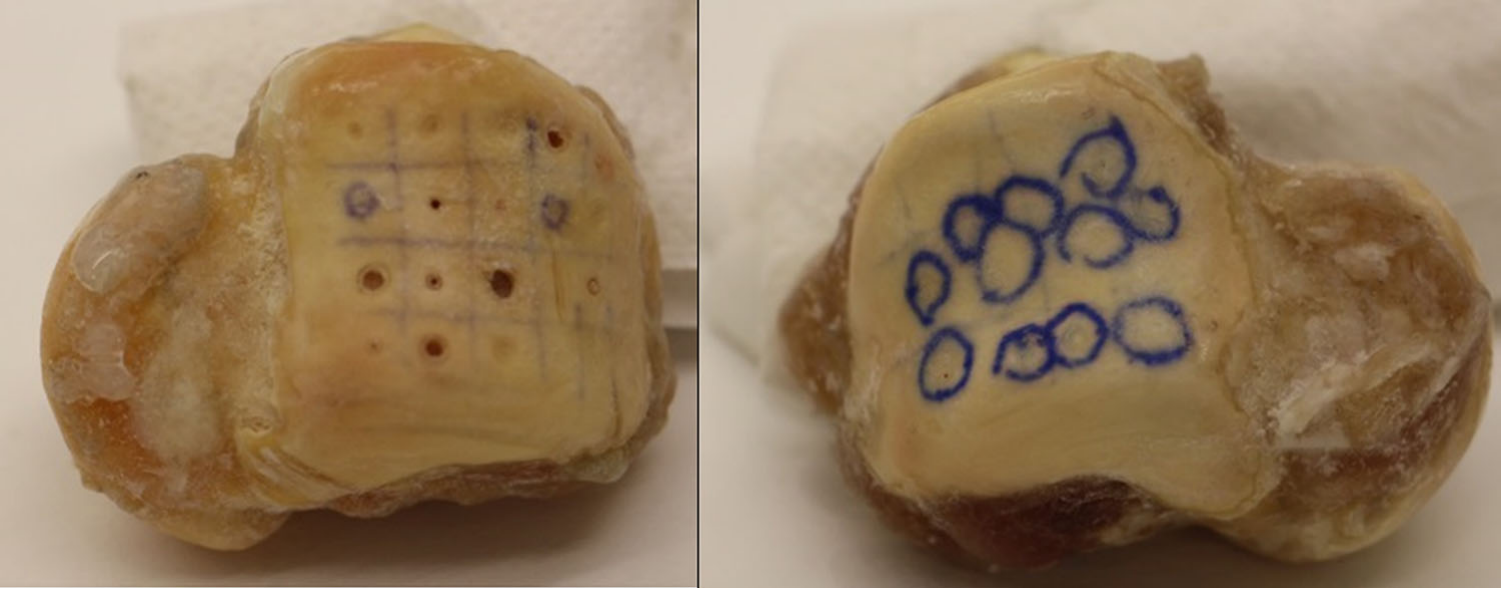
Images of three tali with different types of defects. Left, highlighted are osteochondral and chondral defects (OCD and CD) of various diameters created with drills. Right, chondral surface fibrillation (CSF) inside the marked rings created with sandpaper.

An *in-vitro* study was conducted on human cadaveric ankle specimens. Tali of six different human ankles with no known age that had previously been stored at − 26 °C were prepared to expose the bone and the cartilage surface by removing the soft tissue. The bones were frozen again directly after preparation and thawed an hour before the experiment, during which each specimen was hydrated with saline. Directly afterwards the specimens were frozen and imaged with the µCT scanner on the same day. Three types of cartilage defects were created: osteochondral defects (OCD), chondral defects (CD) and chondral surface fibrillation (CSF) (Fig. 2).

### Creation of Defect Types and Sizes

**Figure 3:**
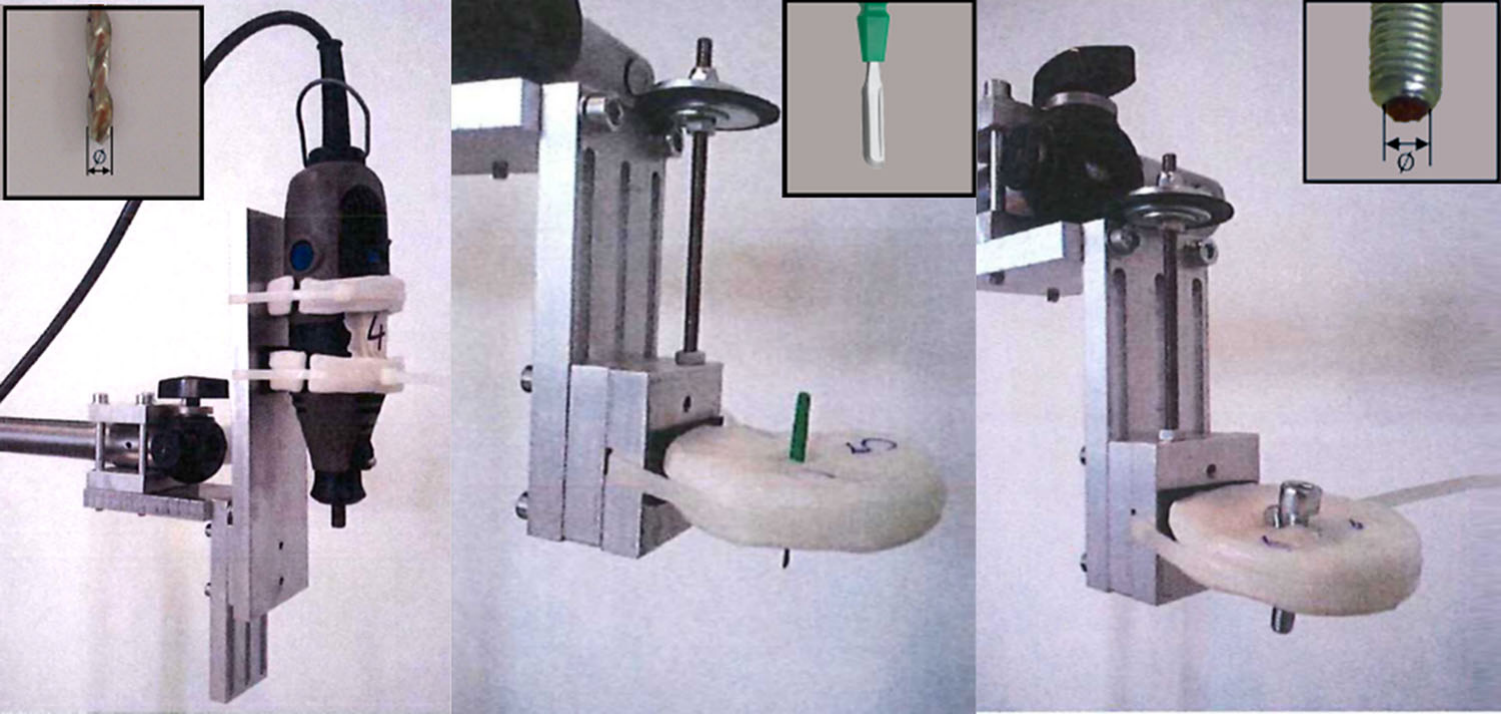
Custom-made holders and plates manufactured to ensure a constant position and orientation above the defect. From left to right: a holder for hand-held drill bits with different diameters; a plastic plate for the Beaver knife; a plastic plate holding one of the three bolts (each bolt has sandpaper with a different diameter glued to the bottom).

**Figure 4:**
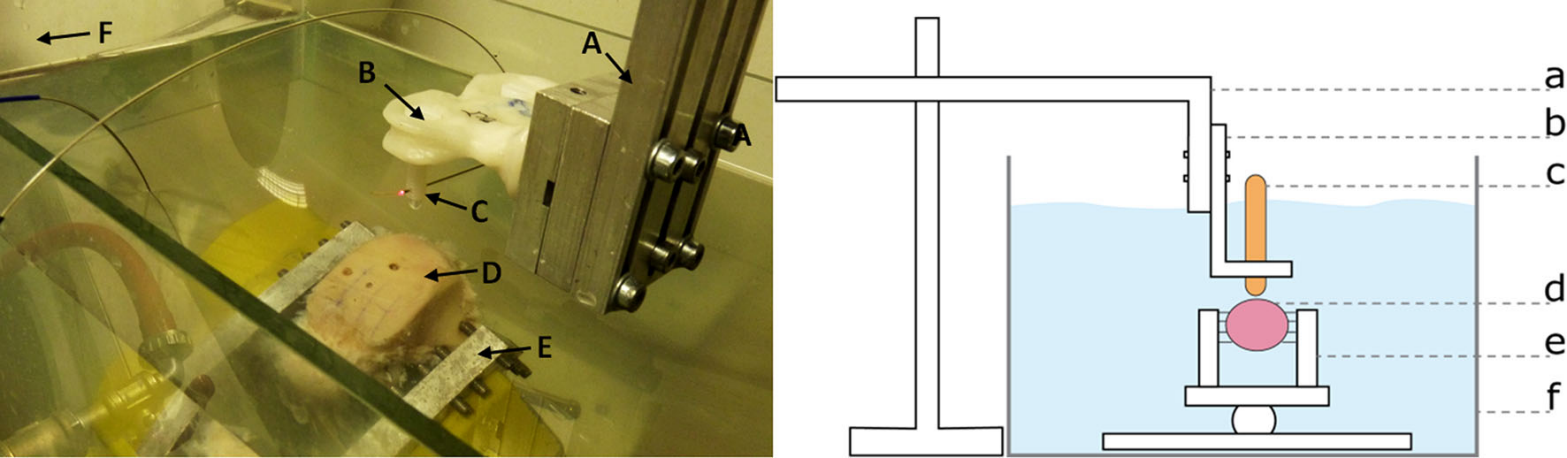
Measurement setup. Left, part of the experimental setup that holds the catheters in a fixed position and orientation above the measurement location on the bone surface. Right, schematic overview of the experimental setup, which consists of a metal frame (A, a) to which clasps (B, b) holding the two bolts and imaging systems (C, c) were attached. The catheter transducers were aligned directly above the specimen. The cadaveric specimen (D, d) was fastened in the specimen vice (E, e) and positioned in a tank filled with saline solution (F, f).

A setup was designed to create each defect type in a standardised manner (Fig. 3). All defects were created perpendicular to the cartilage surface by manual adjustment of the specimen vice under visual inspection (Fig. 4e). OCD and CD defects were created using a hand-operated drill with 0.5, 1, 2 and 3 mm drill bits. The drilling depth was set at either 4 mm to create an OCD or 1 mm to create a CD. A surgical “Beaver” knife was used to create CD 0.1 mm in diameter. CSF was created with 140 grit sandpaper^33^ that was glued to disks 1, 2 and 3 mm in diameter, which were then attached to the bottom of a bolt. The bolt was pressed against the cartilage surface and a manual half turn back and forth of the bolt was repeated five times to create the surface damage. Care was taken to create each defect condition at another location on each specimen to compensate for the effect of site dependence.^9^,^23^,^28^ Other than that, the locations were assigned randomly and each defect condition was created at least six times.

### Measurement Setup

To avoid changes in tissue status due to degradation or unintentional repositioning, the cartilage surface location was measured consecutively by both IVUS and OCT with a custom-made plastic catheter holder that holds the catheter tips in the same position and orientation above the target (Fig. 4, left side). The setup allows the catheter holders to be changed without moving the specimen or setup. The complete measurement setup (Fig. 4, right side) consisted of a robust stainless steel frame (a) to which the plastic plate with bolts (b) was attached. A catheter was inserted through holes in the bolts (c). The frame allows the catheter to be manually adjusted both horizontally and vertically to accurately position it above the target location on the surface. The specimen (d) was fastened in a customised specimen vice (e) that was placed in a glass tank filled with saline solution (f). The specimen vice was equipped with custom-made beaks that contained a grid of sharpened adjustable stainless steel bolts that facilitate firm fixation. The vice’s ball joint allows the alignment of the specimen perpendicular to the catheter and allows the positioning of the cartilage surface as close as possible to the catheter transducer by visual inspection.

### Imaging Systems

Two mobile catheter imaging systems were used: intravascular ultrasound (IVUS) and optical coherence tomography (OCT). The IVUS measurement system consisted of a clinical IVUS main unit (Volcano Corporation, CA, USA) in combination with a Volcano Revolution 45 MHz rotational Imaging Catheter (Volcano Corporation, CA, USA) with a distal outer diameter of 3.2 Fr (~ 1.2 mm) and an intravascular resolution of 50 *µ*m. The automatic pullback system can scan a trajectory of up to 150 mm. The high-resolution IVUS images were captured and stored as 2D images (TIFF format). The OCT imaging system was the C7-XR™ Intravascular Imaging System (St. Jude Medical, Inc., MN, USA), which emits near-infrared light with a data sampling rate of 100 frames per second to produce real-time, ultra-high definition images combined with the Dragonfly™ Imaging Catheter (St. Jude Medical, Inc., MN, USA). This catheter, which has an outer diameter of 2.7 Fr (~ 0.9 mm), produces images with an axial resolution of 15 *μ*m and a lateral resolution of 30–35 *μ*m at 1300 nm.^30^ The automatic pullback system can scan a trajectory of up to 520 mm. For both systems, we set a pullback trajectory of 30 mm, which covered the surface area of the tali. The high-resolution OCT images were captured and stored as 2D images (TIFF format). As reference imaging, µCT scans were made of all prepared specimens before and after creating the defects in air. The µCT scanner (Quantum FX, Perkin Elmer, Waltham, MA) was set with the following parameters: 90 kV tube voltage, 180 *µ*A tube current, 3 min. scan time and isotropic voxel size of 42 × 42 × 42 *µ*m. These settings in combination with scanning in air gave sufficient contrast to image cartilage. The 3D reconstructed images were converted to 2D image of the slices (TIFF format) by using the built-in software of the µCT scanner (Analyze 11.0).

### Experimental Procedure

The six frozen specimens were scanned with the µCT scanner and checked for any existing defects. Each of the defrosted specimens was then fixed in the vice and placed inside the tank. The ball joint of the vice was adjusted to align the selected defect location parallel to the catheter. A single measurement trial was conducted as follows: first, the pre-defect measurements with IVUS and OCT were conducted. Both IVUS and OCT catheters were pulled along a trajectory of 30 mm along the cartilage surface to mimic the intended application in a needle-like instrument. This implied that the distance and orientation between the transducer and cartilage surface could show variations. Second, the defect was created at a predefined location. Third, post-defect measurements were conducted with IVUS and OCT. After this trial, the vice’s ball joint was adjusted so that the next defect location was aligned with the catheter, and the process was repeated until all grid areas on the specimen surface had been used. After all defects had been created and measured with both imaging systems, the specimen was removed from the setup and refrozen. Finally, a second µCT scan was made of all six frozen specimens.

### Image Processing

The reconstructed images of IVUS and OCT were stored without further processing. The open-source image processing software Image J (version 1.50a) was used for image post-processing. The µCT images were reoriented to show the defect using a stack alignment plugin (Align3 TP, version 2010/11/12).^23^ All TIFF stacks were examined and the image that showed the largest defect diameter at the defect bottom was defined as the centre of the defect. The cartilage was inspected adjacent to the defect in the same image to prevent misinterpretation due to unintended cartilage damage of any type. The cartilage thickness of each specimen was measured directly adjacent to each defect at the first location the μCT image slice showed a smooth, undisturbed cartilage layer. The defect diameter and depth values provided by the measurement software were rounded off to the nearest 0.01 mm.

### Drill Hole Visualization Errors

Five different holes (0.1, 0.5, 1, 2 and 3 mm in diameter) were drilled to a depth of 1 mm (CD) and 4 mm (OCD). In this study, µCT was regarded as the gold standard and OCT and IVUS were investigated for their accuracy and sensitivity compared to µCT. By subtracting the measured µCT diameter from the measured OCT and IVUS diameters, a value remains that represents the measured diameter difference (thus OCT–µCT and IVUS–µCT). Similarly, the drill depth measured with µCT was subtracted from the drill depth measured with OCT and IVUS and the difference was used for comparison purposes.

### Statistical Analysis

In the first step of this study, two observers were used to assess each surface defect on the cartilage. The success rate per imaging system was expressed as the percentage of correctly determined defects per type of surface defect (OCD, CD, and CSF). A defect was considered real only when both observers were able to detect it. Its dimensions were then measured during the second step of this study. The sensitivity of the imaging systems were considered equal when an equal number of defects were found within a 95% accuracy. Statistical analyses were performed with IBM SPSS Statistics (v22, IBM Corp., NY, USA) to indicate the presence of any difference in the value of the diameter and insertion depth between the imaging systems compared to the gold standard µCT (i.e., IVUS–µCT vs. OCT–µCT). An *f* test was used to test the null hypothesis that the variances of the populations are equal (*p* > 0.05). In the case of equal variance, a double-sided paired Student’s *t* test was used to test the null hypothesis that the means of two populations are equal. The statistical significance was set at *p* < 0.05.

## Results

**Figure 5:**
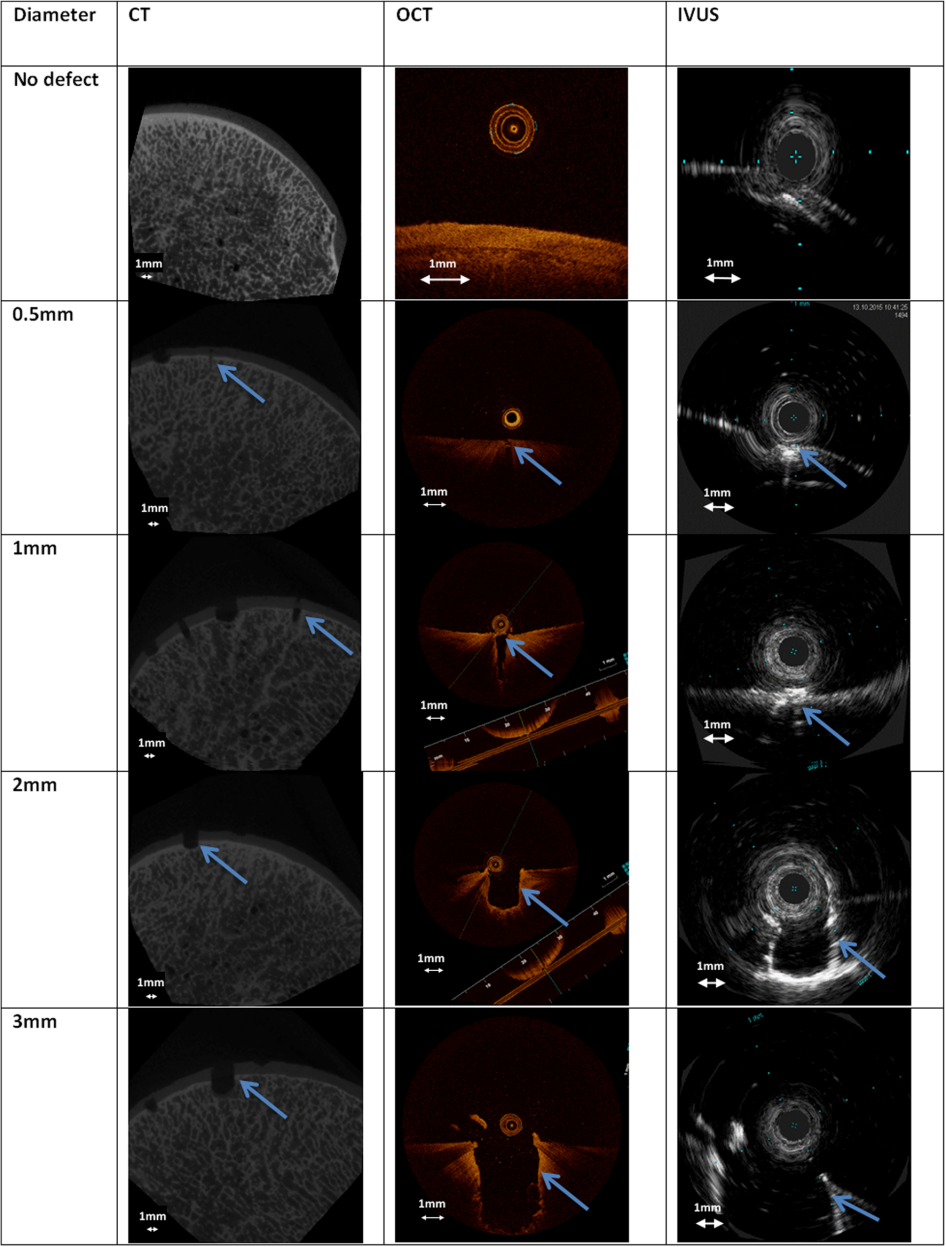
Examples of µCT, OCT and IVUS images from 0.5, 1, 2 and 3 mm osteochondral defects. The arrows indicate the location of the defects.

**Figure 6:**
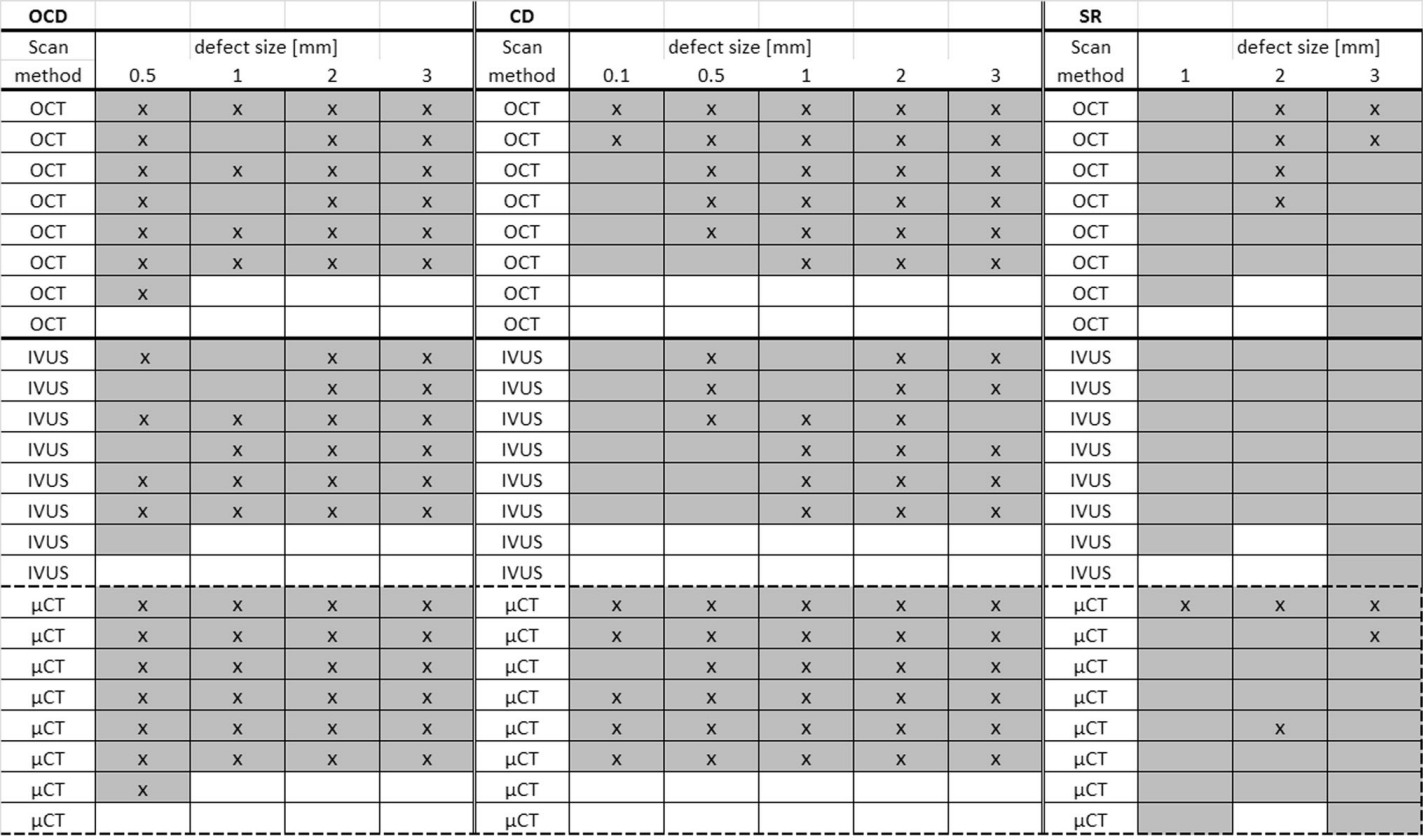
Defects seen and missed by observers specified for each type of defect and imaging method. “X” indicates a defect, indicated by a grey field, seen by both observers. An empty grey field indicates that one or both observers missed the defect.

In total, 25 OCD, 30 CD and 21 CSF defects were created on the six tali. Figure 5 shows a similar sized OCD defect as visualized with the different imaging techniques. Both observers correctly identified the presence of all diameters of OCD defects in 80% of the cases with IVUS, 92% with OCT and 100% with µCT. For all diameters of CD defects, the figures were 60% with IVUS, 83% with OCT and 97% with µCT, and for all diameters of CSF defects, 0% with IVUS, 29% with OCT and 24% with µCT (Fig. 6). The percentage of observed CSF defects was too low and the diameter and depth of this type of damage was too difficult to measure. Therefore, only the OCD and CD defects were further analysed and measured.

**Figure 7:**
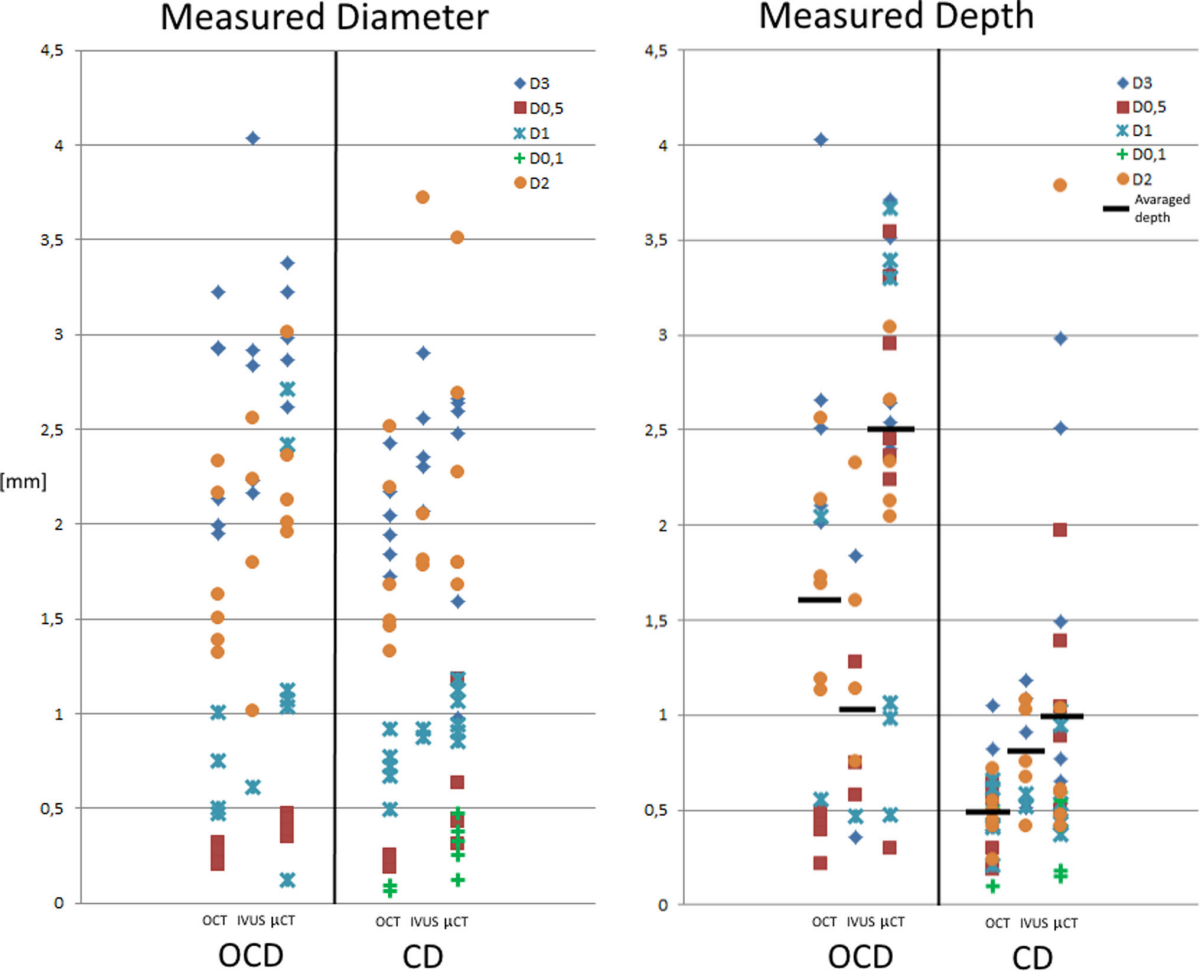
True measured diameter and insertion depth. Left, diameter of holes in OCD and CD measured with OCT, IVUS and µCT. Right, insertion depth of holes in OCD and CD measured with OCT, IVUS and µCT. Drill bits had diameters of 0.1, 0.5, 1, 2 and 3 mm. The aimed for drill depth was 4 mm for OCD and 1 mm for CD.

**Figure 8:**
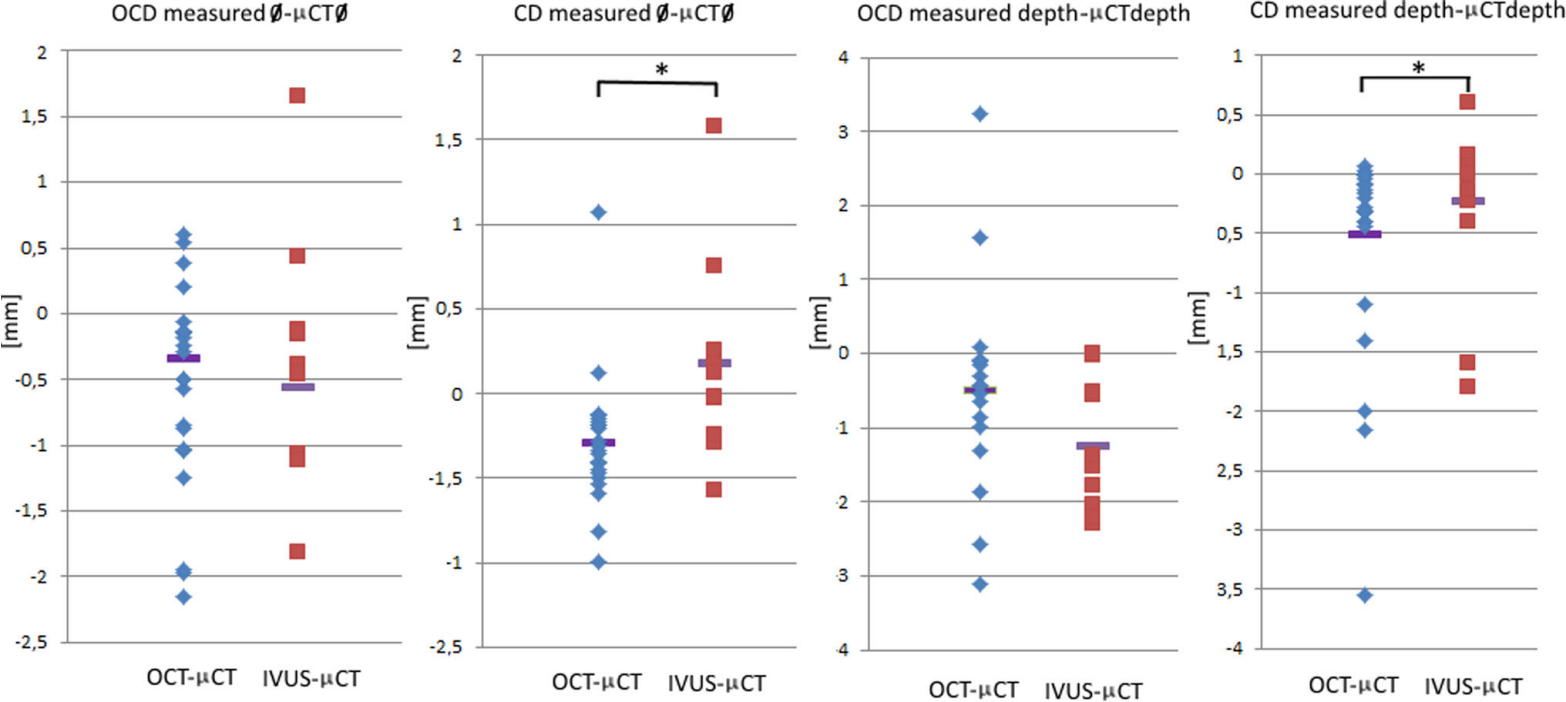
Diameter and insertion depth errors. Left, diameter error (OCT–µCT and IVUS–µCT) of holes in OCD and CD. Right, insertion depth error (OCT–µCT and IVUS–µCT) of holes in OCD and CD. Drill bits had a diameter of 0.1, 0.5, 1, 2 and 3 mm. The aimed for drill depth was 4 mm for OCD and 1 mm for CD. “*” indicates a significant difference between populations.

The cartilage thickness for all specimens was on average 0.9 mm (standard deviation of 0.1 mm). The measured diameter and insertion depth for OCD and CD for IVUS and OCT are shown in Fig. 7. The differences in measured diameter and depth for OCD and CD for OCT–µCT and IVUS–µCT are shown in Fig. 8. The statistical outcomes are presented in Table 1. When testing for equality of variance, no significant difference was found between the imaging systems. Therefore, a Student’s *t* test for equal variance was used for further statistical analysis.

**Table 1:** The outcomes of the *f* test and *t* tests for OCD and CD defect dimension differences in Fig. 7 for each of the imaging methods OCT and IVUS.

Dimension difference	Comparison	Osteochondral defects (OCD)		Chondral defects (CD)	
		*f* test *p* value	*t* test *p* value	*f* test *p* value	*t* test *p* value
Diameter	OCT–µCT vs. IVUS–µCT	0.678	0.591	0.101	0.004
Insertion depth	OCT–µCT vs. IVUS–µCT	0.141	0.333	0.547	0.002

Analysis of the diameter and insertion depth difference for OCD showed no significant difference between OCT–µCT and IVUS–µCT. Analysis of the diameter difference for CD showed a significant difference between OCT–µCT and IVUS–µCT, with an underestimation for OCT and an overestimation for IVUS. Analysis of the insertion depth difference for CD showed a significant difference between OCT–µCT and IVUS–µCT, both giving an underestimation, with OCT giving a lower value.

## Discussion

With the introduction of injectable hydrogels, the need has arisen for new needle-like instruments to allow early-stage interventions for small cartilage defects in joints. Such a new type of steerable needle is under development (Fig. 1). To allow the correct localisation of the needle tip on top of the isolated damaged cartilage prior to injection and sealing with a hydrogel, we studied two catheter imaging systems for their suitability for integration in the slender steerable needle. The detection rate of OCT and IVUS was measured compared to the gold standard µCT, and it was at least 80% for OCD and 60% for CD (Fig. 6) for diameters ranging from 0.5 to 3 mm, which were smaller than previously tested.^6^,^33^ OCT and IVUS are depth-limited techniques where the depth of penetration varies from 1–2 mm for OCT to 4–8 mm for IVUS. The achievable resolution with OCT is 10–20 *µ*m whereas that for IVUS is 100–150 *µ*m.^24^

The results indicates that the thin talar cartilage regions can be successfully visualised by both techniques. The detection rate was higher for OCT compared to IVUS, especially when the defects were smaller and less deep, because the IVUS resolution limits its detection rate. This was also confirmed by the data given in Fig. 7, which indicate that it was not possible to measure the detected 0.1 and 0.5 mm diameter holes with IVUS. The higher detection rate in combination with the smaller diameter of the OCT catheter makes OCT the preferred candidate for integration in needle-like hydrogel delivery devices to be used for OCD and CD defect types (Fig. 1).

The detection rate of CSF was rather low for all imaging systems (Fig. 6). This is in contrast to earlier studies by Viren^33^ and by Saarakkala *et al*.^27^ In-depth analysis indicates that this is predominantly caused by the differences in study design: rather old, previously frozen human bones with a thin cartilage layer vs. fresh animal bones with a thick cartilage layer, fine grinding (140 grit) vs. course grinding (60 grit), small defect size (< Ø 3 mm) vs. large defect size (> Ø 5 mm), variation in distance vs. optimal constant distance to the surface, B-mode analysis vs. ToF signal analysis. Furthermore, during this study, the bone specimens underwent multiple freeze–thaw cycles for logistic reasons. We expect that this marginally influenced the morphology of the bone and cartilage defects. As this was a predominant outcome parameter that influenced all conditions equally, the influence on the relative comparison of the two imaging systems can be considered negligible. However, further studies are needed to quantify the true influence of freeze–thaw cycles, as this limitation makes comparison with other studies such as Viren^33^ and Saarakkala^27^ difficult.

We also processed a single measurement per condition vs. taking the average of up to 10 measurements in the other studies.^33^ The latter approach obviously increases the signal-to-noise ratio and enhances the image quality of CSF for both OCT and IVUS. IVUS images show quite some noise, which is typical in sound reflection imaging modalities (Fig. 4). It could be that the CSF was performed too gently without causing enough macroscopic structural cartilage disruption and that the noise is in the same range as that caused by CSF. In retrospect, we could have made arthroscopic or microscopic photographic images to assist the µCT recordings. On the other hand, these differences indicate the contribution of our study. Our study design of taking a single measurement and using a pullback trajectory that allows the distance between the transducer and cartilage surface to vary, mimics more closely the actual clinical practice and the intended manual steering of the foreseen needle-like instrument. Apparently, this does imply that certain defect types and sizes can no longer be detected.

In the present study, outcome measures were based on geometrical differences rather than full tissue-quality characterisation. The dimensions of the defects can be accurately assessed using the photographic images of IVUS and OCT. Furthermore, µCT is known to be superior in detecting bony tissue and has the advantage of providing 3D geometrical information as opposed to histology.^22^ In this sense, the use of µCT as the gold standard is defendable, especially to image cartilage, as we verified that cartilage could be seen with our scanning protocol in air. The latter is also confirmed by the cartilage thickness measurements that we performed, which are in line with the results of other studies.^1^,^3^,^18^ Contrast-enhanced CT using Hexabrix or CA4 + or histology could be used in future studies to assess the cartilage quality.^35^ Finally, histological analysis of multiple sections taken from and around the defect sites could have highlighted more details on site dependence and cartilage composition.

Although OCT has a 46% higher success rate than IVUS when detecting CSF defects, it is unlikely that the steerable needle device will be used for the treatment of surface fibrillation with hydrogel as there is not much space to fill. Thus, OCT imaging applied in the proposed application provides a promise for the future of early-stage defect detection and intervention in a one-stop solution for patients who have sustained a trauma known to have a high likelihood of cartilage damage.

Although both catheter imaging techniques allow the measurement of defect diameters and depths for OCD and CD, the results presented in Fig. 7 indicate that the measured values can deviate by twice the actual diameter and depth as defined by the drill bits that were used to create the defects. One reason for this is that the composition of cartilage and bone material did not allow the creation of holes with higher accuracy. Another reason is that the small OCD and CD defects were in the spatial resolution range of all three imaging modalities and the thickness of one trabecula (ca. 0.3 mm). Therefore, just penetrating a trabecula wall (wall thickness of around 0.05 mm) with the drill tip can result in a depth measurement error of up to 0.3 mm, as the open space in the trabecula is possibly added to the drill depth during the measurement.^10^ Finally, the cylindrical 2D images from the catheters do show some errors due to catheter surface debris (Fig. 5, 0.5 mm IVUS), non-uniform rotation distortion (NURD) effects (Fig. 5, 0.5 and 2 mm IVUS), and reflection and refraction caused by the harder bone that result in artefacts and deformation, which makes it more difficult to estimate the dimensions.^31^ Therefore, further experiments should also include a focus on improvements in catheter purging, catheter tip actuation and optimisation of the distance between surface and catheter.

Figure 7 shows that a mean difference of 0.6 mm was found for the estimated defect depth of 4 mm deep holes between IVUS and OCT, showing that IVUS has more difficulty in assessing the correct depth. However, comparing the mean insertion depth difference for the 4 mm deep hole of both catheter imaging techniques with the gold standard (1.1 mm IVUS and 1.6 mm OCT vs. 2.5 mm µCT) indicates that neither imaging technique should be used to determine the depths of deeper holes. For clinical application, the found accuracy and variance are acceptable, because for the intended application an estimate of the volume of the defect is relevant to deciding the volume of hydrogel that should be injected, but this could also be verified real-time when the catheter is present.

In the present study, a method was employed that uses an automated retraction mechanism to scan a particular area of an open joint. In order to investigate its applicability in a more realistic setting, a closed joint study should be performed to evaluate the ease of use of the total needle imaging concept. In the presented setup, the distance between scan area and catheter is fixed. In order to determine the accuracy of the images in a real-life setup, off-centre tests and the creation of accuracy plots vs. distance from the centre should be investigated in a series of additional experiments. As catheter holders were interchanged during the experiments, a lot of effort was made to standardize the experiment with a strong focus on repeatability. However, due to some tolerances in the components, it is still possible that not exactly the same slice was taken when making the images with each different imaging method. To reduce this risk in further experiments, it is advised to use small metal markers as references.

### Future Work

Previous studies where OCT catheters have been manually inserted into joints^7^ showed that manoeuvring the catheter inside the joint can be challenging. To investigate whether OCT catheters have a minimally invasive clinical application in orthopaedics, the next step would be to implement the OCT catheter in the design shown in Fig. 1, followed by a series of cadaver experiments conducted with orthopaedic surgeons to investigate the practical aspects of this combined device. In the last phase of this research, the reliability of this kind of system inside and outside the hospital should be determined.

## Conclusion

Mechanically created defects as small as 0.1 mm can be detected with an OCT catheter in *in-vitro* human talar cartilage and bone, making it the preferred candidate for future integration in needle-like instruments for the precise localisation of the instrument relative to pathologic tissue.
